# Hepatitis C virus infection: a challenge in the complex management of two cases of multidrug-resistant tuberculosis

**DOI:** 10.1186/s12879-019-4494-1

**Published:** 2019-10-22

**Authors:** Maria Musso, Silvia Mosti, Gina Gualano, Paola Mencarini, Rocco Urso, Piero Ghirga, Alessia Rianda, Franca Del Nonno, Delia Goletti, Fabrizio Palmieri

**Affiliations:** 1 Respiratory Infectious Diseases Unit, National Institute for Infectious Diseases “L. Spallanzani” IRCCS, Rome, Italy; 2 Hepatology Unit, National Institute for Infectious Diseases “L. Spallanzani” IRCCS, Rome, Italy; 3 Pathology Unit, National Institute for Infectious Diseases “L. Spallanzani” IRCCS, Rome, Italy; 4 Translational Research Unit, National Institute for Infectious Diseases “L. Spallanzani” IRCCS, Rome, Italy

**Keywords:** Multidrug-resistant tuberculosis, Chronic hepatitis C, Treatment, Drug-induced liver injury

## Abstract

**Background:**

Multidrug-resistant tuberculosis (MDR-TB) requires lengthy use of second-line drugs, burdened by many side effects. Hepatitis C virus (HCV) chronic infection increases risk of drug-induced liver injury (DILI) in these patients. Data on MDR-TB patients with concurrent HCV chronic infection treated at the same time with second-line antitubercular drugs and new direct-acting antivirals (DAAs) are lacking. We evaluate if treating at the same time HCV infection and pulmonary MDR-TB is feasible and effective.

**Cases presentation:**

In this study, we described two cases of patients with pulmonary MDR-TB and concurrent HCV chronic infection cured with DAAs at a Tertiary Infectious Diseases Hospital in Italy. During antitubercular treatment, both patients experienced a DILI before treating HCV infection.

After DAAs liver enzymes normalized and HCV RNA was undetectable. Then antitubercular regimen was started according to the institutional protocol, drawn up following WHO MDR-TB guidelines. It was completed without further liver side effects and patients were declared cured from both HCV infection and MDR-TB.

**Conclusions:**

We suggest to consider treatment of chronic hepatitis C with DAAs as a useful intervention for reintroduction of second-line antitubercular agents in those patients who developed DILI, reducing the risk of treatment interruption when re-exposed to these drugs.

## Background

Treatment of multidrug-resistant tuberculosis (MDR-TB) requires second-line anti-TB drugs that are more costly, less efficacious and more toxic than first-line drugs [1].

Most patients on treatment for MDR-TB, lasting up to 2 years, experience at least one adverse drug reaction that can lead to the interruption of treatment and contribute to unfavorable outcome [2].

Drug-induced liver injury (DILI) refers to a hepatic injury due to a medication, herb or dietary supplement [3]. It is one of the most frequent adverse drug reactions related to antitubercular drugs [4] and his spectrum of injury ranges from asymptomatic liver tests elevation to acute liver failure [3].

DILI can result from several second-line drugs used in MDR-TB regimen [5–8].

Hepatitis C virus (HCV) chronic infection prevalence is more elevated in people affected by TB than in general population [9] and it is a well-known independent risk factor for the development of drug induced hepatotoxicity [5, 8]. HCV chronic infection makes the already complex management of MDR-TB patients even more difficult.

New direct-acting antivirals (DAAs) changed chronic hepatitis C from a barely manageable to a curable condition. No clear recommendations for MDR-TB patients with concurrent HCV chronic infection treated at the same time with second-line antitubercular drugs and DAAs are currently available.

After we obtained informed consent from patients, we report two cases of successful treatment of pulmonary MDR-TB and concurrent HCV chronic infection cured with DAAs.

## Cases description

### Case 1

A 39 years old woman of Romanian origin affected by retreated pulmonary MDR-TB with resistance to isoniazid, rifampin, pyrazinamide, streptomycin, amikacin and kanamycin (proportion method in Lowenstein-Jensen medium) and HCV chronic infection was referred to our Institute. Before starting antitubercular treatment liver function tests were normal. She has been treated for 3 months with (daily dosage, unless otherwise specified): levofloxacin (1000 mg), cycloserine (750 mg), para-aminosalicylic acid (8 g), linezolid (600 mg), ethionamide (750 mg), and bedaquiline (200 mg three times per week). At admission to our Institute, she complained nausea, asthenia and loss of appetite. Severe liver enzymes alteration (AST/ALT =113/284 U/L) was found. HCV viral load was 253.336 IU/ml, genotype 1b. Additional causes of liver injury have been excluded. According to the institutional protocol, drawn up following WHO MDR-TB guidelines [10], treatment was interrupted.

Liver biopsy performed for staging chronic hepatitis documented mild necrotizing and inflammatory activity and portal fibrosis, grade 5 (A2 + B0 + C1 + D2) and stage 2 of Ishak score, grade A1 (PMN1 + LN0) and stage F1 of METAVIR score [11, 12]. Sofosbuvir/ledipasvir 400/90 mg once daily was started.

**Fig. 1:**
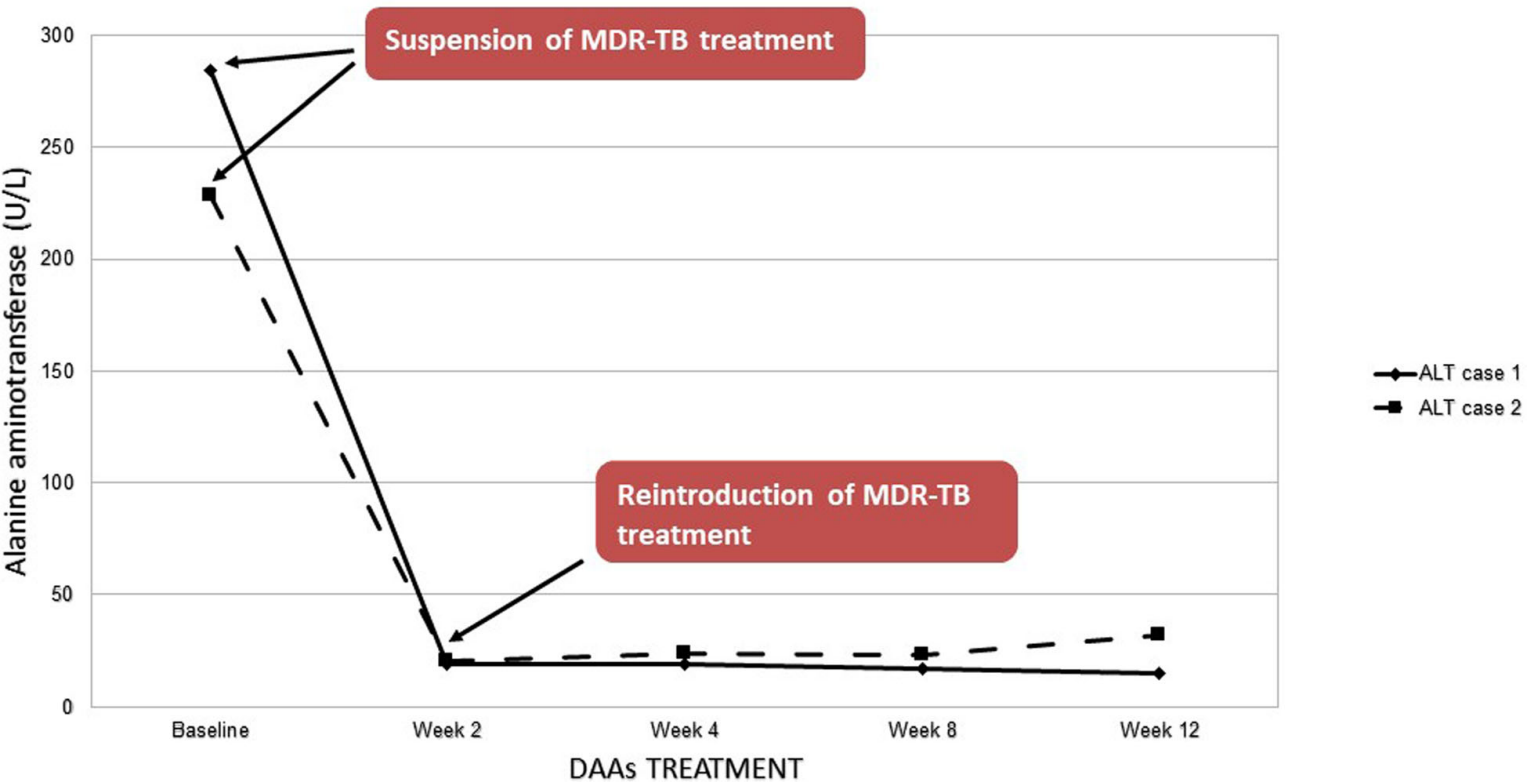
Pattern of alanine aminotrasferase values during DAAs therapy and after reintroduction of antitubercular treatment. Legend. DAAs: Directly Acting Antivirals, ALT: Alanine aminotransferase

After 2 weeks liver enzymes normalized and HCV RNA was undetectable (Fig. 1). Then an individualized regimen according to drug sensitivity test results and current WHO guidelines, adjusted for comorbidities, was restarted with moxifloxacin (400 mg), cycloserine, linezolid, ethionamide, ethambutol (1200 mg), and clofazimine (100 mg) [10].

A sustained HCV virological response was reached after 12 weeks of DAAs and cure according WHO guidelines [10] was obtained after 20 months of treatment without other hepatic adverse drug events. HCV viral load was still suppressed after 7 months of follow up.

### Case 2

A 66 years old man from Ethiopia was referred with diagnosis of MDR-TB. His clinical history included only HCV chronic infection. Aspartate aminotransferase (AST) and alanine aminotransferase (ALT) were normal (< 40 U/L) at admission in our Institute (HCV RNA was 32.874 IU/ml, genotype 4d).

Mycobacteria growth indicator tube on liquid media (MGIT 960 systems; Becton Dickinson, Sparks, MD, USA) detected resistance to rifampin and isoniazid. A regimen with amikacin (1 g), pyrazinamide (2 g), levofloxacin (1 g), linezolid (600 mg), ethambutol (1600 mg), prothionamide (750 mg), and imipenem (2 g)/clavulanic acid (375 mg) was started according to WHO guidelines [10]. At the forth month of treatment nausea and vomit occurred and because of a severe liver enzymes alteration (AST/ALT = 374/228 U/L) regimen was interrupted.

HCV RNA increased to 463.428 IU/ml. Additional causes of liver injury have been excluded. A liver biopsy documented a grade 9 (A3 + B2 + C2 + D2), stage 4 of Ishak score and grade A2 (PMN2 + LN1), F2 of Metavir score [11, 12].

Velpatasvir/sofosbuvir 100 mg/400 mg per day was prescribed.

Normalization of transaminases occurred after 2 weeks and regimen with amikacin, levofloxacin, linezolid, ethambutol, prothionamide, imipenem/clavulanic acid, and cycloserine (750 mg) was restarted (Fig. 1).

HCV viral load was suppressed at 12 weeks of DAAs treatment.

Patient was declared cured after 20 months of antitubercular treatment without other hepatic adverse drug events. HCV viral load is still suppressed at sixth months of follow up.

## Discussion and conclusions

We report two cases of successful treatment of pulmonary MDR-TB and concurrent HCV chronic infection cured with DAAs. Treatment of MDR-TB is challenging because it relies on drugs with lower efficacy and much greater toxicity than those used for drug-susceptible TB (DS-TB) [13].

Treatment outcomes for MDR-TB and extensively drug-resistant TB (XDR TB) are generally poor if compared to DS-TB [14]. Adverse drug reactions including DILI represent an obstacle to treatment completion and may negatively influence the outcome of MDR-TB [15].

Documented liver toxicity has been described for several drugs used in MDR-TB regimens like pyrazinamide, ethionamide/prothionamide cycloserine, clofazimine, para-aminosalicylic acid, linezolid, bedaquiline, delamanid, and moxifloxacin [5–8, 16]. According to available data, patients receiving MDR TB treatment require drug suspension from the regimen due to adverse reactions in 30% of cases and treatment interruption in 2.1% [2]. Risk of DILI in patients with tuberculosis ranges in several studies from 5% to as high as 33% of treated cases, with a rate of asymptomatic increase of liver enzymes around 20% [16]. DILI is not uncommon in MDR-TB treatment and the incidence reported is usually around 2%, but a pick of 16.8% has been described [2, 8, 17–19].

During antitubercular treatment DILI is more common in patients infected by hepatotropic viruses [18]. HCV infection represents a significant risk factor for DILI in MDR-TB treatment [18], but it seems not related with outcome of these patients [18, 20].

Assessment of liver dysfunction or biliary disease, use of alcohol and assumption of any hepatotoxic drugs are mandatory at diagnosis of TB and screening for viral hepatitis should be considered for patients with risk factors [16].

Pharmaceutic armamentarium in MDR-TB is limited and includes drugs with potential liver toxicity. Reducing the cumulative risk of hepatic side effects in these patients by treating HCV chronic infection is crucial in order to complete a long and challenging therapeutic regimen.

Data on co-administration of DAAs and second-line anti TB drugs are lacking. No clear recommendations for MDR-TB patients with concurrent HCV chronic infection treated at the same time with second-line antitubercular drugs and DAAs are currently available.

In low TB burden countries management of MDR-TB patients should be performed in referral centers in order to ensure active TB drugs safety monitoring and timely management of adverse events and co-morbidities [21].

Treatment with DAAs allowed to reintroduce successfully an effective second-line antitubercular treatment without recurrence of DILI in our two cases.

HCV chronic infection is an additional challenge for clinicians in the already complex management of MDR-TB.

As far as we know these are the first two case reports of a successful concurrent treatment of MDR-TB and HCV chronic infection with second-line antitubercular drugs and DAAs in a low TB incidence country like Italy, but further investigations by clinical trials would be warranted to evaluate this treatment strategy.

Treatment with DAAs is feasible and should be considered in MDR-TB patients with HCV chronic infection. This permits to complete challenging antitubercular regimen in condition of multidrug resistance using also second-line potentially hepatotoxic drugs and minimizing the risk of prolonged treatment interruption.

## Data Availability

All data are presented in this manuscript and can be accessed through the corresponding author on request.

## Abbreviations

ALT: Alanine transaminase
AST: Aspartate transaminase
DAAs: Direct-acting antivirals
DILI: Drug-induced liver injury
DS-TB: Drug-susceptible TB
HCV: Hepatitis C virus
MDR-TB: Multidrug-resistant tuberculosis
XDR-TB: Extensively drug-resistant TB
